# Modified bone marrow mesenchymal stem cells derived exosomes loaded with MiRNA ameliorates non‐small cell lung cancer

**DOI:** 10.1111/jcmm.70115

**Published:** 2024-09-25

**Authors:** Mingjun Yang, Wen Zhou, Xiao Han, Mingming Xu, Zhipeng Wang, Min Shi, Yanyan Shi, Yunchi Yu

**Affiliations:** ^1^ Department of Cardiothoracic Surgery Affiliated Hospital of Nantong University Nantong Jiangsu China; ^2^ Department of Thoracic Surgery Haimen People's Hospital Nantong Jiangsu China

**Keywords:** exosomes, LXY30 peptide, miR‐30c, miR‐181b and miR‐613, non‐small cell lung cancer

## Abstract

The study aimed to reveal the function of LXY30 peptide‐modified bone marrow mesenchymal stem cell‐derived exosomes (LXY30‐Exos) in NSCLC. LXY30 peptide is a peptide ligand targeting α3β1 integrin, and LXY30 specifically binds to Exos derived from different cells. We use transmission electron microscopy to identify LXY30‐Exos and tracking analysis for particles, and the LXY30‐Exos internalized by NSCLC cells in vitro and targeted NSCLC tumours in vivo were verified by multiple molecular technologies. The functions of LXY30‐Exos‐encapsulated miR‐30c, miR‐181b or miR‐613 were assessed using cell proliferation, migration and cell apoptosis assays. Meanwhile, the safety of the above engineered Exos was evaluated in vivo. After LXY30‐Exos were isolated and identified, LXY30‐Exos were confirmed to be internalized by NSCLC cells in vitro and specifically targeted NSCLC tumours in vivo. Functionally, LXY30‐Exos‐encapsulated miR‐30c, miR‐181b or miR‐613 weakened the proliferation, migration and cell cycle of NSCLC cells induced cellular apoptosis in vitro and restrained the tumour progression in vivo. Meanwhile, the safety of LXY30‐Exos‐encapsulated miR‐30c, miR‐181b or miR‐613 was confirmed in vivo. Overall, miR‐30c, miR‐181b and miR‐613 encapsulated in LXY30 peptide‐modified BMSC‐Exos relieved NSCLC.

## 1 INTRODUCTION

More than 85% of lung cancer cases worldwide are non‐small cell lung cancer (NSCLC).^1^, ^2^ Despite advancements in surgery and chemotherapy, the 5‐year survival rate for NSCLC patient's remains low, highlighting the need for improved strategies to enhance clinical outcomes. Conventional cancer chemotherapy and radiotherapy have serious side effects, development of drug resistance and cross‐resistance, cancer migration and recurrence (PMID: 25470047). Current treatments, including targeted therapies and immunotherapies, have shown promise; Further research on this basis is urgently needed to overcome resistance mechanisms, target different tumour subtypes and provide better tolerance to patients.

Exosomes (Exos) are small extracellular vesicles, and the diameter of Exos is 30–150 nm.^3^ Exos act as a medium of intercellular communication by transporting proteins, lipids, microRNAs (miRNAs) and other active biomolecules between cells.^4^ Exos are released from bone marrow mesenchymal stem cells (BMSCs) under specific physiological or pathological conditions.^5^, ^6^ Cumulative studies authenticate that BMSCs‐derived Exos (BMSC‐Exos) exert momentous regulatory functions in NSCLC development.^7^, ^8^ Among these investigations, the function of BMSC‐Exos in NSCLC via the transport of miRNAs has attracted much attention. For instance, BMSC‐Exos decreases the cisplatin resistance of NSCLC cells by transporting miR‐193a.^9^ Exosomal miR‐144 from BMSCs represses NSCLC cell proliferation and colony formation.^10^ As Exos research deepens, cancer therapies based on Exosomal miRNAs are being developed. Genetic tools are used to enhance the cell‐targeting properties of Exos, containing receptors or antibodies against tumour biomarkers.^11^, ^12^ Inspired by this, accumulated studies have focused on using these properties to design engineered Exos to deliver miRNAs to specific tumour tissues.^13^, ^14^ However, the explicit targeting of NSCLC by miRNAs packaged in engineered Exos remains to be investigated further.

Integrins are 24 heterodimer somatic surface receptors, they are involved in cell adhesion, tumorigenesis and a novel tumour biomarker and drug target.^15^ Integrin α3β1 is a receptor for laminin, collagen, fibronectin, chondroitin sulfate proteoglycan 4 and epiligrin.^16^ Abnormally high expression of α3β1 integrin is closely interrelated to tumorigenesis and poor prognosis of NSCLC.^17^, ^18^ LXY30 is a cyclic peptide and is the ligand for α3β1 integrin's α3 subunit.^19^ LXY30 has been explained to bind to α3 integrin of NSCLC cells.^20^ Thus, LXY30 is a suitable peptide ligand targeting α3β1 integrin in NSCLC.^21^ Previous studies demonstrate that miR‐30c, miR‐181b and miR‐613 exert the tumour‐suppressive functions in NSCLC.^22^, ^23^, ^24^ Therefore, we attempted to elucidate the function of engineered Exos encapsulated miR‐30c, miR‐181b or miR‐613 in NSCLC and to tentatively evaluate its safety, which may provide an effective strategy for NSCLC treatment.

## 2 RESULTS

### 2.1 Extraction and characterization of LXY30 peptide‐modified Exosomes

**FIGURE 1:**
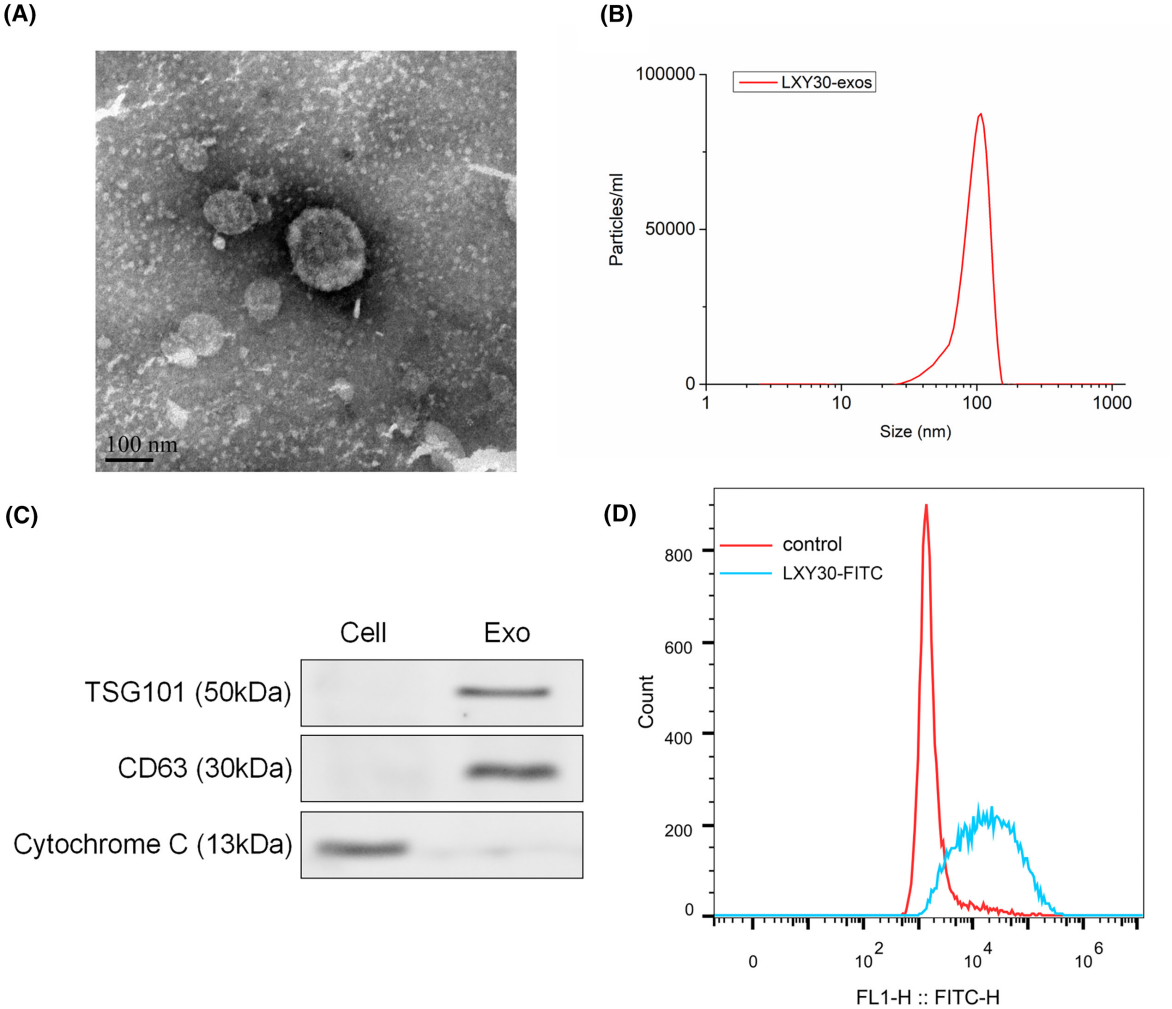
Isolation and characterization of LXY30 peptide‐modified exosomes. (A, B) LXY30 peptide‐modified BMSC‐Exos (LXY30‐Exos) were isolated. The morphology and size of LXY30‐Exos were evaluated using transmission electron microscopy (TEM, scale bar: 100 nm) and nanoparticle tracking analysis (NTA). (C) The protein levels of the Exos markers TSG101, CD63, and Cytochrome C were determined by Western blot. (D) After the A549 cells were harvested, the cells were incubated with LXY30‐FITC for 2 h. The specific binding of LXY30 was assessed using flow cytometry. Exo, exosome; FITC, Fluorescein isothiocyanate.

Previous studies authenticate that LXY30 peptide binds to α3β1 integrin on cancer cells to perform its tumour‐targeting function.^20^, ^25^ Thus, this research attempted to elucidate the LXY30 peptide‐modified BMSC‐Exos (LXY30‐Exos) function in NSCLC. After the LXY30‐Exos were extracted, the morphology and size of the Exos were identified (Figure 1A,B).

TSG101 and CD63 are common positive markers for Exos, and Cytochrome C is negative.^26^, ^27^ As illustrated in Figure 1C, TSG101 and CD63 are highly expressed in LXY30‐Exos, while Cytochrome C is not. Meanwhile, flow cytometry results revealed that A549 cells were specifically bound to LXY30‐FITC (Figure 1D). Therefore, these results support the successful isolation of LXY30‐Exos and the specific binding of LXY30‐FITC to NSCLC cells.

### 2.2 Engineered Exos are internalized by NSCLC cells

**FIGURE 2:**
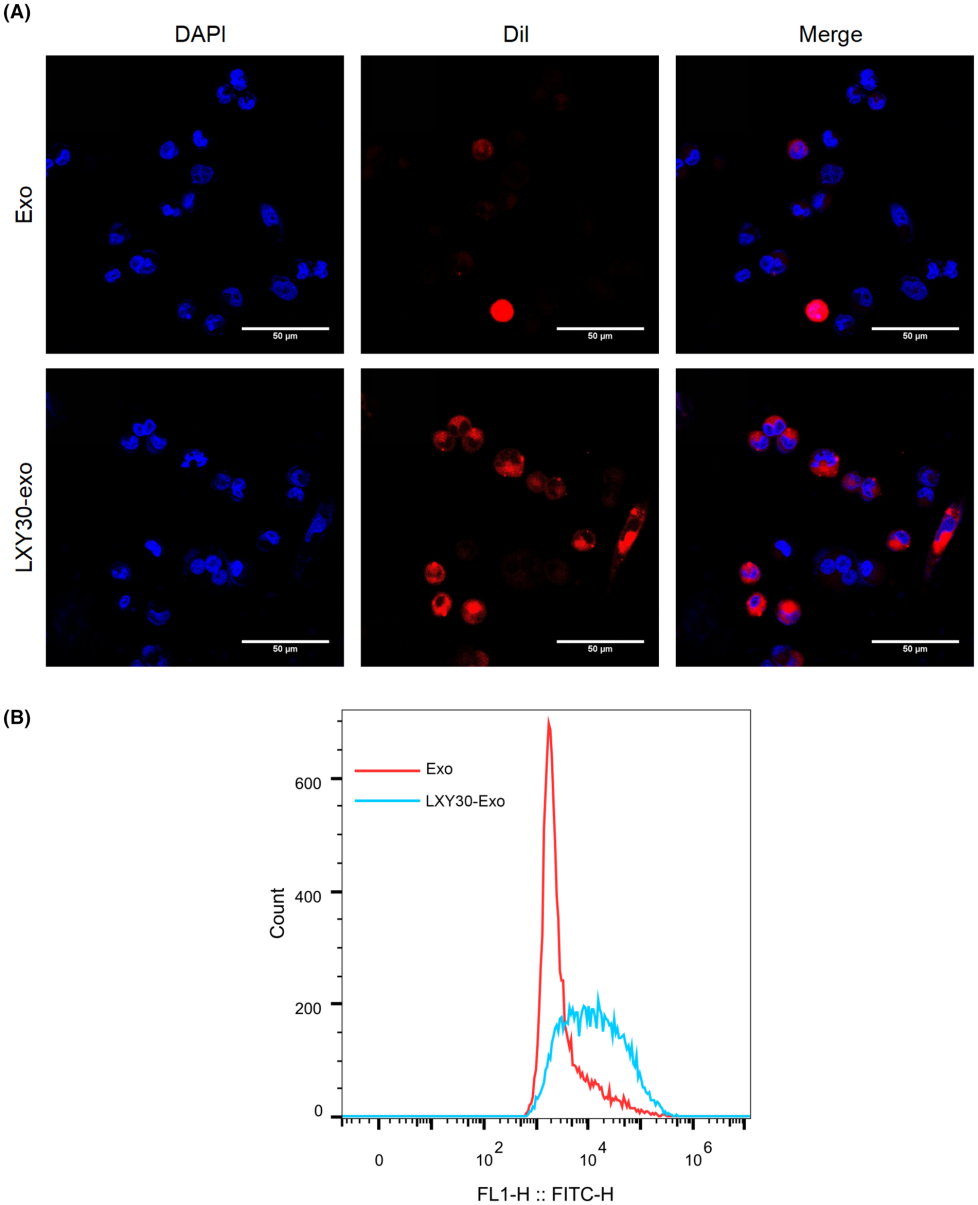
Uptake of LXY30‐Exos by NSCLC cells. (A) Verification of Dil‐labelled LXY30‐Exos (red) was internalized by DAPI‐labelled A549 cells (blue) by a confocal laser scanning microscope (scale bar: 50 μm). (B) The uptake of LXY30‐Exos by A549 cells was confirmed using flow cytometry.

LXY30 binds to α3 integrin on NSCLC cells and enters cells through endocytosis.^20^ Therefore, we further investigated whether LXY30‐Exos could be ingested by NSCLC cells. Dil‐labelled LXY30‐Exos were co‐cultured with DAPI‐labelled A549 cells. As exhibited in Figure 2A, more red fluorescence signals were detected in the cytoplasm of cells in LXY30‐Exos than in Exos. Furthermore, the uptake of Exos by A549 cells was assessed, and the results clarified that the fluorescence intensity of LXY30‐Exos was higher than that of Exos, implying that more LXY30‐Exos entered into A549 cells (Figure 2B). Generally, LXY30‐Exos could be internalized by NSCLC cells.

### 2.3 Engineered Exos target NSCLC tumours

**FIGURE 3:**
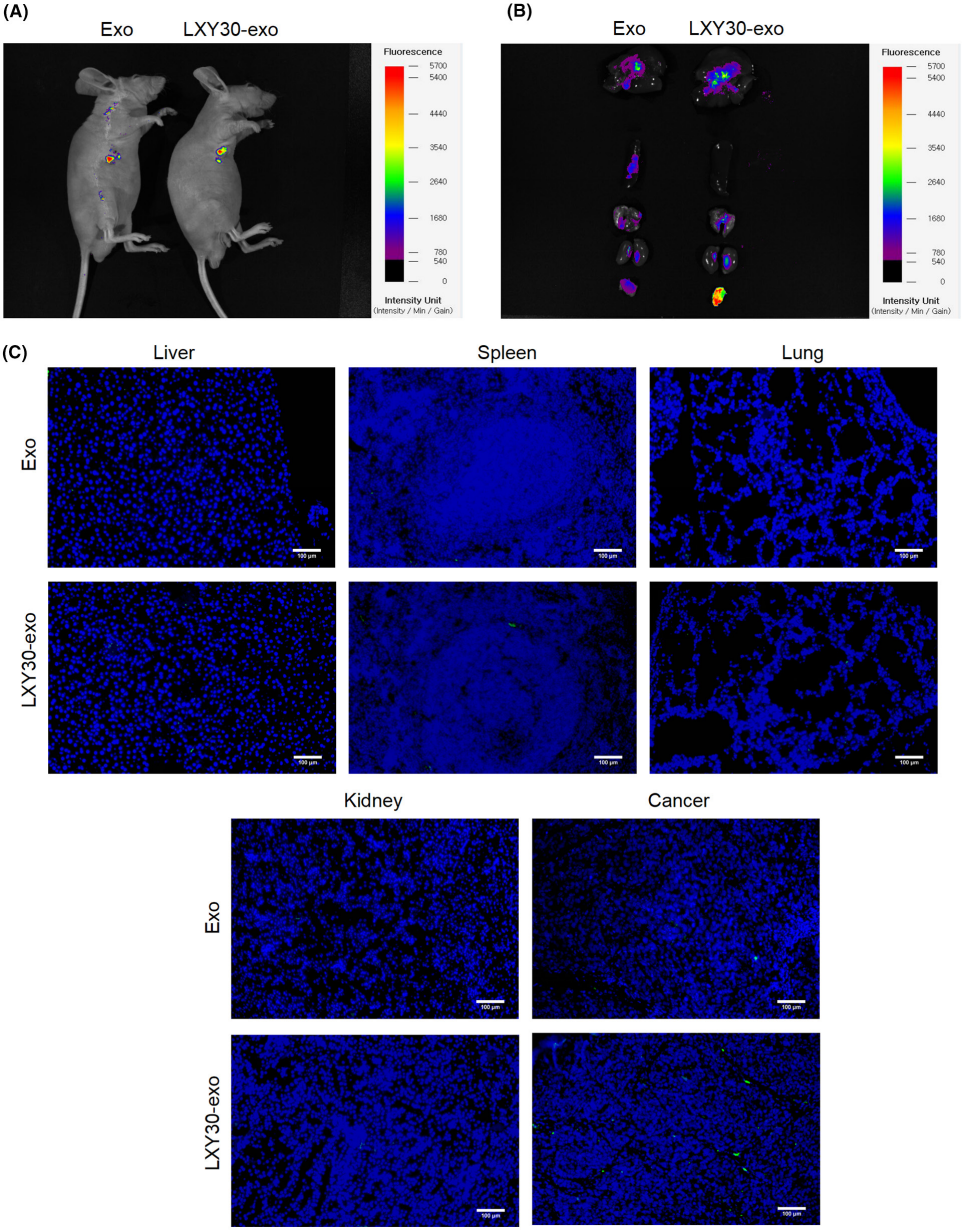
Validation of the in vivo tumour‐targeting of LXY30‐Exos. NSCLC xenograft model was constructed, and PKH67‐labelled Exos and PKH67‐labelled LXY30‐Exos were intraperitoneally injected into mice. (A) In vivo, imaging analysis was performed 21 days after injection. (B) The fluorescence intensity of liver, spleen, lung, kidney, and tumour tissues was determined using fluorescent staining. (C) Comparison of PKH67‐labelled Exos in liver, spleen, lung, kidney, and tumour tissue sections (scale bar: 100 μm). Green, PKH67‐labelled Exos or PKH67‐labelled LXY30‐Exos; Blue, DAPI.

To determine whether Exos are effective for delivery, it is critical to understand their tumour targeting and in vivo distribution. PKH67 is usually applied to label Exos.^28^ After the establishing of the NSCLC xenograft model, PKH67‐labelled LXY30‐Exos were injected into mice. As shown in Figure 3A, the relative fluorescence of the tumour region was higher in LXY30‐Exos than in Exos. Moreover, fluorescent staining also revealed that the fluorescence of the tumour region was elevated in LXY30‐Exos in comparison with Exos, but there were no remarkable changes in the fluorescence intensity of liver, spleen, lung and kidney tissues (Figure 3B). Meanwhile, more PKH67‐labelled LXY30‐Exos were observed in tumour tissue sections (Figure 3C). Concluding, LXY30‐Exos targets NSCLC tumours.

### 2.4 Engineered Exos encapsulated‐miRNAs repress NSCLC cell proliferation and migration

Previous studies demonstrate that miR‐30c, miR‐181b and miR‐613 exert tumour‐suppressive functions in NSCLC.^22^, ^23^, ^24^ Similarly, we confirmed the low expressions of miR‐30c, miR‐181b and miR‐613 in NSCLC clinical samples and cell lines (Figure S1A,B). Meanwhile, we clarified that miR‐30c, miR‐181b and miR‐613 were efficiently transferred into LXY30‐Exos (Figure S1C). The LXY30‐Exos‐encapsulated miR‐30c (Exo‐miR‐30c), LXY30‐Exos‐encapsulated miR‐181b (Exo‐miR‐181b) or LXY30‐Exos‐encapsulated miR‐613 (Exo‐miR‐613) were co‐cultured with A549 cells. As displayed in Figure S1D, miR‐30c, miR‐181b and miR‐613 were highly expressed in A549 cells, prompting that A549 cells to absorb either Exo‐miR‐30c, Exo‐miR‐181b or Exo‐miR‐613.

**FIGURE 4:**
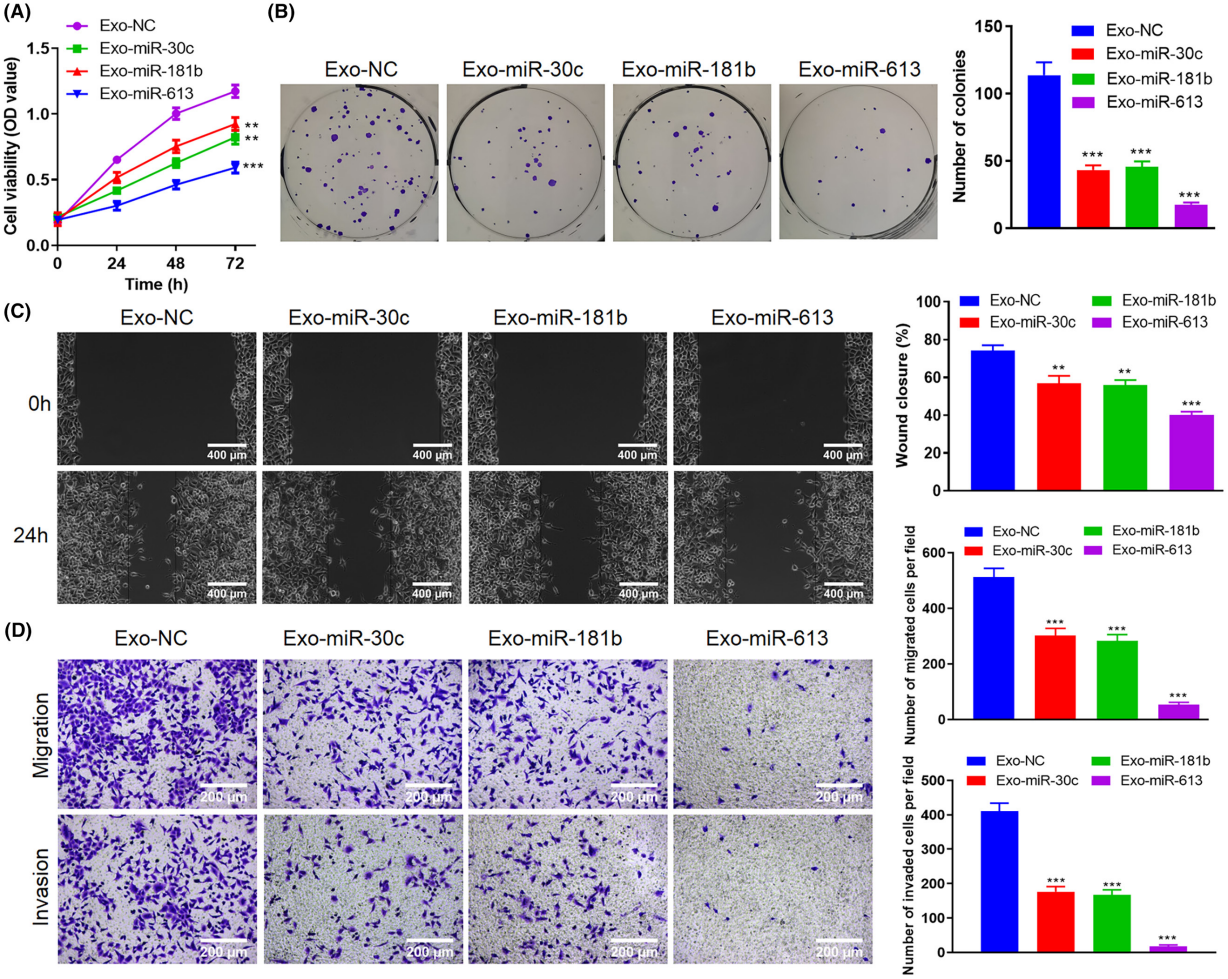
Engineered Exos encapsulated‐miRNAs regulate the NSCLC cell proliferation and migration. LXY30‐Exos‐encapsulated miR‐30c (Exo‐miR‐30c), LXY30‐Exos‐encapsulated miR‐181b (Exo‐miR‐181b), or LXY30‐Exos‐encapsulated miR‐613 (Exo‐miR‐613) were co‐cultured with NSCLC cells for 24 h. (A) NSCLC cell proliferation was assessed using a Cell Counting Kit‐8 (CCK‐8) assay. (B) Comparison of NSCLC cell proliferation by colony formation assay. (C, D) The NSCLC cell migration was determined using Wound healing (scale bar: 400 μm) and Transwell assays (scale bar: 200 μm). **p < 0.01, ***p < 0.001 versus Exo‐NC. NC, negative control.

Based on the above findings, we further evaluated the Exo‐miR‐30c, Exo‐miR‐181b and Exo‐miR‐613 impact on the A549 cell proliferation and migration. As exhibited in Figure 4A, the Exo‐miR‐30c, Exo‐miR‐181b and Exo‐miR‐613 weakened the A549 cell proliferation compared to Exo‐NC. Similar to this conclusion, the colony formation assay further confirmed that compared with Exo‐NC, the A549 cell proliferation was repressed in Exo‐miR‐30c, Exo‐miR‐181b and Exo‐miR‐613 (Figure 4B). Meanwhile, the Exo‐miR‐30c, Exo‐miR‐181b and Exo‐miR‐613 restrained the A549 cell migration (Figure 4C). Transwell assay further verified that the A549 cell migration was reduced in Exo‐miR‐30c, Exo‐miR‐181b and Exo‐miR‐613 compared to Exo‐NC (Figure 4D). In conclusion, the engineered Exos encapsulating miR‐30c, miR‐181b or miR‐613 repressed the NSCLC cell proliferation and migration.

### 2.5 Engineered Exos encapsulated‐miRNAs induce NSCLC cell cycle arrest and facilitate cell apoptosis

**FIGURE 5:**
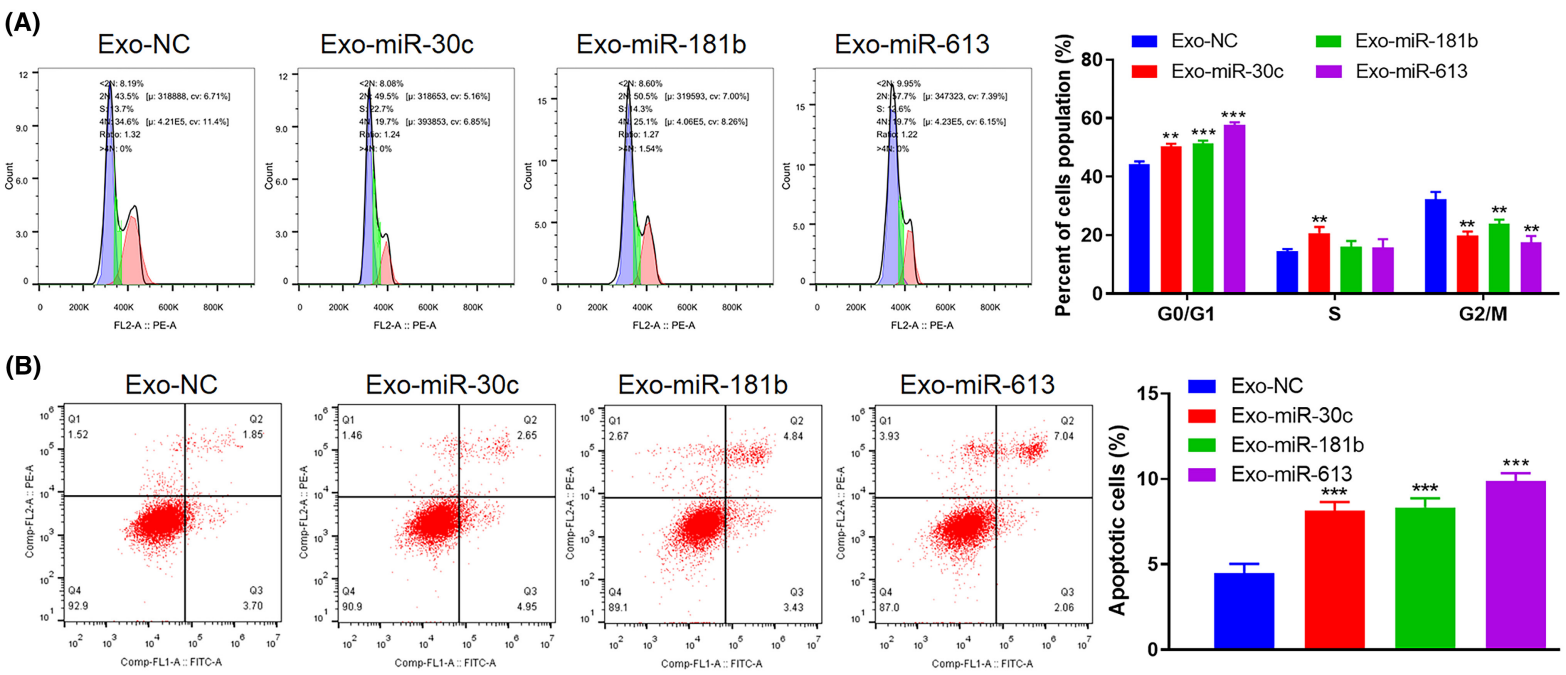
Engineered Exos encapsulated‐miRNAs influence the cell cycle and apoptosis of NSCLC cells. Exo‐miR‐30c, Exo‐miR‐181b, and Exo‐miR‐613 were co‐cultured with A549 cells for 24 h. (A) The cell cycle of A549 cells was assessed using flow cytometry. (B) Analysis of A549 cell apoptosis by flow cytometry. **p < 0.01, ***p < 0.001 versus Exo‐NC.

We further assessed the effects of Exo‐miR‐30c, Exo‐miR‐181b and Exo‐miR‐613 on the regulation of the NSCLC cell cycle and apoptosis. As illustrated in Figure 5A, compared with Exo‐NC, Exo‐miR‐30c, Exo‐miR‐181b or Exo‐miR‐613 induced the A549 cell cycle arrest compared to Exo‐NC. Meanwhile, the A549 cell apoptosis was assessed, and the results clarified that Exo‐miR‐30c, Exo‐miR‐181b and Exo‐miR‐613 accelerated the A549 cell apoptosis (Figure 5B). All in all, the engineered Exos encapsulated miR‐30c, miR‐181b or miR‐613 induced NSCLC cell cycle arrest and accelerated cell apoptosis.

### 2.6 Engineered Exos encapsulated‐miRNAs inhibit tumour growth in vivo

**FIGURE 6:**
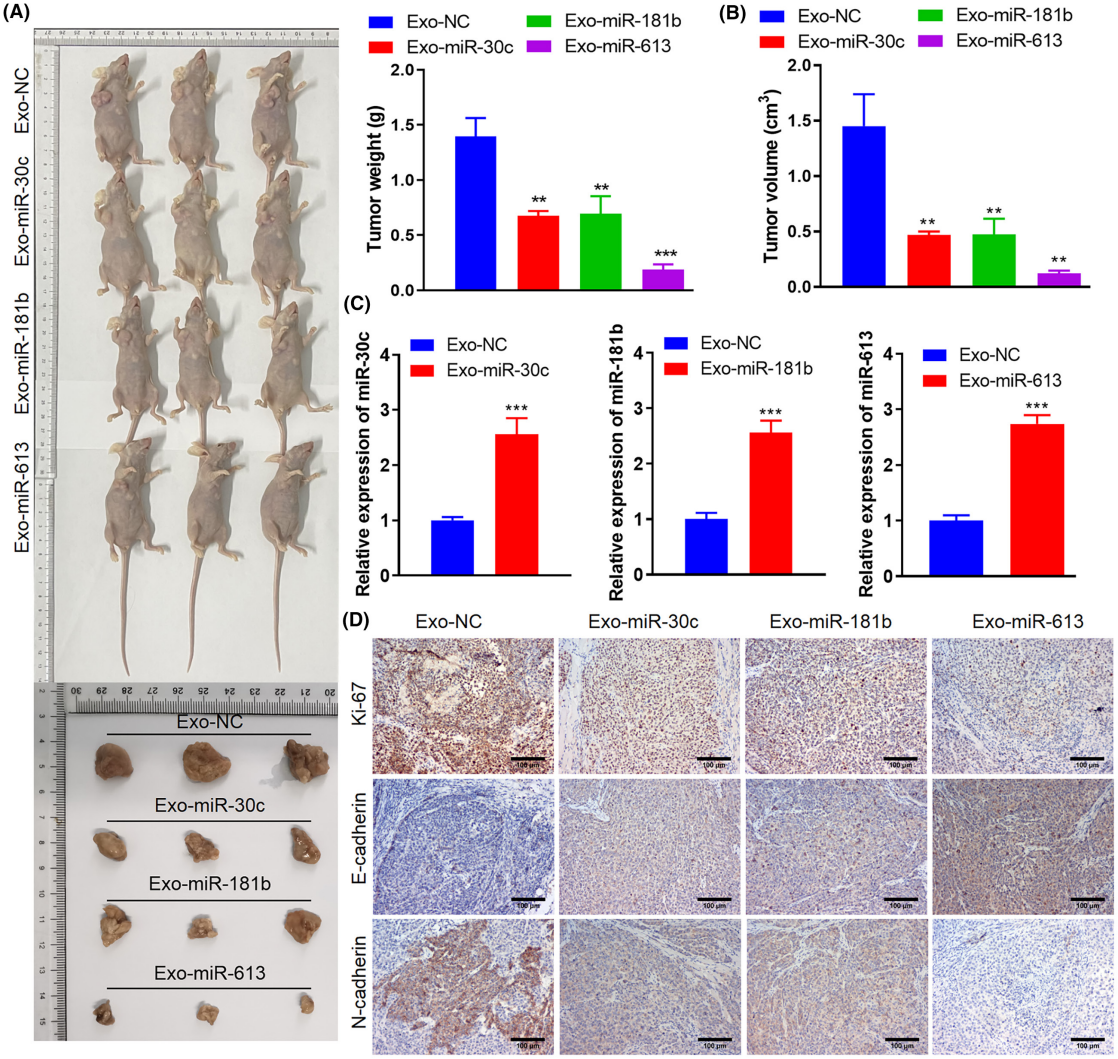
Engineered Exos encapsulated‐miRNAs influence tumour growth in vivo. The NSCLC xenograft model was constructed and Exo‐miR‐30c, Exo‐miR‐181b and Exo‐miR‐613 were injected into mice intraperitoneally. (A) In vivo imaging analysis was performed 14 days after the injection, and the tumour weight was measured. (B) The tumour growth curve and the tumour volume were calculated. (C) Analysis of the miR‐30c, miR‐181b, and miR‐613 expressions in NSCLC tissues using quantitative real‐time PCR (qRT‐PCR). (D) The Ki‐67, E‐cadherin, and N‐cadherin expressions were determined using an immunohistochemical assay (scale bar: 100 μm). **p < 0.01, ***p < 0.001 versus Exo‐NC.

Having obtained the protective function of engineered Exos encapsulated miRNAs in the in vitro NSCLC model, we further investigated their role in the in vivo NSCLC model. As illustrated in Figure 6A,B, the injection of Exo‐miR‐30c, Exo‐miR‐181b and Exo‐miR‐613 reduced the tumour weight and volume. Moreover, we evaluated the miRNA expressions in NSCLC tissues, and the findings expounded that miR‐30c, miR‐181b and miR‐613 were upregulated in NSCLC tissues (Figure 6C).

Ki‐67 is a marker for proliferation,^29^ and E‐cadherin and N‐cadherin are markers for epithelial‐mesenchymal transformation and participate in tumour metastasis.^30^, ^31^ Immunohistochemical assays confirmed that the Ki‐67 and N‐cadherin were poorly expressed after the injection of Exo‐miR‐30c, Exo‐miR‐181b or Exo‐miR‐613. In contrast, E‐cadherin was highly expressed, implying that Exo‐miR‐30c, Exo‐miR‐181b and Exo‐miR‐613 reduced the proliferation and epithelial‐mesenchymal transformation (Figure 6D). In short, engineered Exos encapsulated miRNAs inhibited tumour growth in vivo.

### 2.7 Safety evaluation of engineered Exos

**FIGURE 7:**
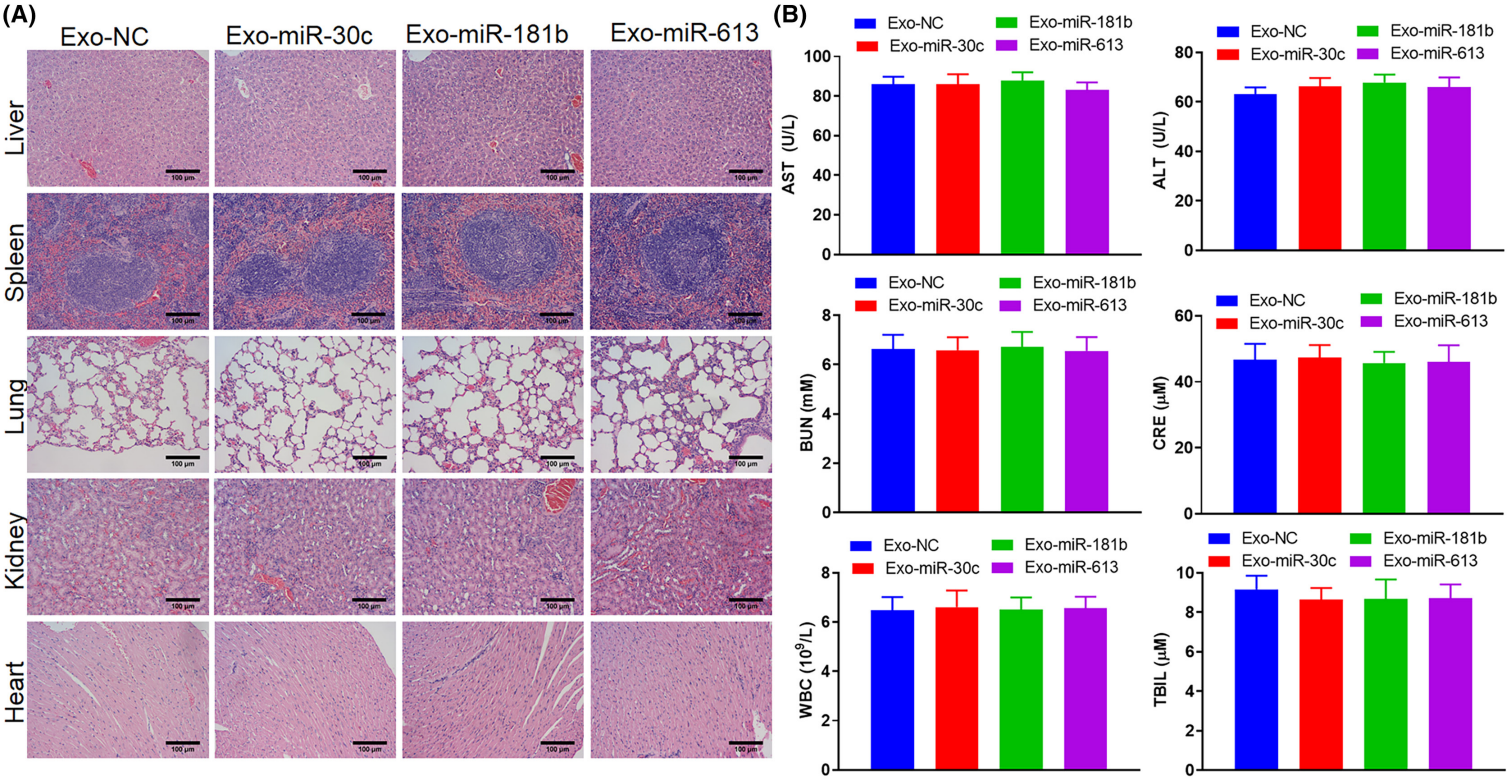
Safety analysis of engineered Exos. NSCLC xenograft model was constructed, and Exo‐miR‐30c, Exo‐miR‐181b, and Exo‐miR‐613 were intraperitoneally injected into mice. (A) The pathological changes of liver, spleen, lung, kidney, and heart tissues were evaluated using HE staining (scale bar: 100 μm). (B) The contents of aminotransferase (AST), alanine aminotransferase (ALT), blood urea nitrogen (BUN), creatinine (CRE), white blood cell (WBC), and total bilirubin (T‐Bil) were measured by enzyme‐linked immunosorbent assay (ELISA).

Having demonstrated the remission of engineered Exos in both in vitro and in vivo NSCLC models, we then assessed the safety of engineered Exos. As illustrated in Figure 7A, the spleen, liver and heart tissues showed no apparent lesions after the injection of Exo‐miR‐30c, Exo‐miR‐181b or Exo‐miR‐613.

Aminotransferase (AST), alanine aminotransferase (ALT), total bilirubin (T‐Bil) are serum biochemical parameters,^32^ blood urea nitrogen (BUN), creatinine (CRE) and white blood cell count (WBC) are routine biochemical indexes.^33^ ELISA results confirmed that compared with Exo‐NC, the contents of AST, ALT, BUN, CRE, WBC and T‐Bil had no noticeable changes after the injection of Exo‐miR‐30c, Exo‐miR‐181b and Exo‐miR‐613 (Figure 7B). All the above studies confirmed the safety of engineered Exos.

## 3 DISCUSSION

NSCLC is a malignant tumour worldwide and still lacks effective treatment strategies.^34^ This study primarily evaluated the function of LXY30 peptide‐modified BMSC‐Exos in NSCLC. First, LXY30 peptide modified BMSC‐Exos was isolated and identified. Second, BMSC‐Exos with LXY30 peptide modification was internalized by NSCLC cells in vitro and targeted to NSCLC tumours in vivo. Furthermore, LXY30‐Exos‐encapsulated miR‐30c (Exo‐miR‐30c), LXY30‐Exos‐encapsulated miR‐181b (Exo‐miR‐181b) or LXY30‐Exos‐encapsulated miR‐613 (Exo‐miR‐613) repressed NSCLC growth in both in vitro and in vivo models. At the same time, the safety of these engineered Exos was confirmed in vivo.

Exos is a promising system that has recently emerged to deliver nucleic acids to treat NSCLC.^35^, ^36^ Exos extracted from BMSCs transfected with miRNAs are a promising treatment option for NSCLC.^9^ For the target specificity, a momentous choice is to use ligands to modify its specific affinity for membrane proteins overexpressed on the tumour cell surface.^37^ α3β1 integrin is a novel type of tumour biomarker molecule, and the elevated α3β1 integrin in tumour cells mediates tumour invasion and metastasis.^38^, ^39^ LXY30 targets α3β1 integrin, and LXY30 prominently increases tumour targeting against ovarian, breast and NSCLC cells.^20^ Crucially, LXY30 binds to NSCLC cells, and Exos derived from different cells.^21^ Drawing on prior research, we successfully isolated and characterized LXY30 peptide‐modified BMSC‐Exos. Subsequent validation confirmed the internalization of these exosomes by NSCLC cells in vitro, as well as their specific targeting of NSCLC tumours in vivo. These findings suggest a potential mechanism by which cellular internalization promotes the generation and release of exosomes.

Exos participate in the regulation of NSCLC by carrying various biologically active molecules, of which miRNAs are the primary mediator.^40^, ^41^ Previous studies illustrate that miR‐30c, miR‐181b and miR‐613 are abnormally expressed in NSCLC and are potential markers for NSCLC.^23^, ^24^, ^42^ Similarly, our study confirmed that miR‐30c, miR‐181b and miR‐613 were low‐expressed in NSCLC clinical samples and cells. One of the concerns of applying source cells to encapsulate miRNAs into secreted Exos is the loading efficiency.^43^, ^44^ Thus, we additionally corroborate that miR‐30c, miR‐181b and miR‐613 were efficiently transferred to LXY30 peptide‐modified BMSC‐Exos, and NSCLC cells took up these engineered Exos. Subsequently, we furthermore evaluated the Exo‐miR‐30c, Exo‐miR‐181b and Exo‐miR‐613 functions in NSCLC. As expected, the engineered Exos encapsulated miR‐30c, miR‐181b or miR‐613 diminished NSCLC cell proliferation, migration and cell cycle progression, facilitated cell apoptosis in vitro, and restrained tumour progression in vivo.

In addition to therapeutic efficacy, toxicity is another critical parameter for additional engineered Exos.^37^ From this, we initially assessed whether engineered Exos were toxic in the in vivo NSCLC model. The data authenticated that mice's liver, spleen, lung, kidney and heart tissues displayed no remarkable lesions after the injection of Exo‐miR‐30c, Exo‐miR‐181b or Exo‐miR‐613. Meanwhile, the AST, ALT, BUN, CRE, WBC and T‐Bil contents had no prominent changes after the injection of Exo‐miR‐30c, Exo‐miR‐181b or Exo‐miR‐613, prompting that these engineered Exos were safe in NSCLC.

Despite the promising results obtained in both in vitro and in vivo models, the translation of this therapy to clinical applications presents several challenges. First, the scalability and reproducibility of producing engineered exosomes with consistent quality and functionality need to be addressed. Large‐scale production of exosomes while maintaining their integrity, purity and targeting ability can be technically challenging. Another challenge is the delivery and biodistribution of these engineered exosomes in humans. While our study demonstrated effective targeting to NSCLC tumours in a murine model, differences in human physiology may affect the exosomes' distribution and targeting efficiency. Potential off‐target effects and the immune response to exogenously administered exosomes also warrant thorough investigation to ensure safety and minimize adverse effects.

In conclusion, while the engineered exosomes encapsulated with miR‐30c, miR‐181b or miR‐613 show substantial promise for NSCLC treatment, overcoming these translational challenges will be crucial for their successful clinical application.

## 4 MATERIALS AND METHODS

### 4.1 Cell culture

BMSCs were procured from Oricell (Guangzhou, China). The cells were grown in Dulbecco's modified Eagle's medium (DMEM)/F‐12 (Gibco, Carlsbad, USA) containing 100 μg/mL streptomycin and 100 U/mL penicillin (Gibco) and 10% fetal bovine serum (FBS, Gibco).

Human NSCLC cell lines (A549 and NCI‐H358) and human normal lung epithelial cells BEAS‐2B were from the American Type Culture Collection (ATCC, Maryland, USA). They were grown in DMEM with 10% FBS 100 U/mL penicillin, and 100 μg/mL streptomycin. Cells were maintained at 37°C with 5% CO_2_.

### 4.2 Extraction and identification of LXY30 peptide‐modified BMSC‐Exos

The BMSC‐Exos extraction was conducted following the previously reported methods.^45^ First, the BMSC cell culture supernatant that contained Exos was collected. The supernatant was first centrifuged at 300 × g for 10 min followed by 1500 × g for 15 min to remove the cells. Subsequently, the supernatant was centrifuged at 10000 × g for 25 min to remove cell fragments. We collect the supernatant and then centrifuge at 100000 × g for 1 h. The microspheres pellet containing Exos were harvested. All centrifugations were conducted at 4°C, and equivalent samples were filtered by 0.22 μm filters (Beyotime Biotechnology, Shanghai, China). Lastly, the pellet was re‐suspended in phosphate‐buffered saline (PBS) (Thermo Fisher Scientific, Massachusetts, USA), and the isolated Exos were BMSC‐Exos.

Exos‐bead binding assay was carried out referring to the previous literature.^21^ BMSC‐Exos (200 μL) was added to the tube containing 100 TentaGel beads coated with LXY30 (SythBio, Anhui, China) and incubated at 37°C for 1 h. The labelled Exos were purified from free peptides using a centrifugal spin column (Thermo Fisher Scientific). LXY30 peptide‐modified BMSC‐Exos (LXY30‐Exos) were identified by Exosomal markers TSG101, CD63 and Cytochrome C.

The synthesis of LXY30‐FITC was carried out by referring to the previous literature.^20^ Subsequently, the 3 × 10^4^ A549 cells were incubated with 0.5 μM LXY30‐FITC for 2 h. Then we used the flow cytometer for the LXY30 binding specificity.

### 4.3 Nanoparticle‐tracking analysis and transmission electron microscopy

In nanoparticle‐tracking analysis (NTA) analysis, the NanoSight LM 10 system equipped with rapid video capture and particle tracking software (NanoSight, UK) is used to analyse exosomes. Data analysis is performed using NTA 2.3 software (NanoSight, UK).

For transmission electron microscopy (TEM) detection, exosomes suspended in 200 μL of PBS are fixed with 4% paraformaldehyde (PFA) and 4% glutaraldehyde in 0.1 M phosphate buffer (pH 7.4) and kept at 4°C. Each exosome sample is placed on a carbon‐coated copper grid, immersed in 2% phosphotungstic acid solution (pH 7.0) for 30 s, and then observed and photographed using a transmission electron microscope (JEOL‐JEM1400, Tokyo, Japan) with an acceleration voltage of 80 kV.

### 4.4 Western blot

Protein concentrations were evaluated using an Enhanced BCA Protein Assay Kit (Beyotime Biotechnology). proteins were extracted from the cells and Exos with an extraction buffer (Sigma‐Aldrich, Missouri, USA). Equal amounts of total protein (25 μg) was separated using 10% SDS‐PAGE gel electrophoresis (Beyotime Biotechnology) and subsequently transferred onto polyvinylidene fluoride membranes (Millipore, Massachusetts, USA). To block non‐specific sites on the membrane were blocked by 5% bovine serum albumin (BSA, Beyotime Biotechnology), then they were incubated with specific antibodies containing TSG101 (ab125011, 1/1000), CD63 (ab134045, 1/2000), and cytochrome C (ab133504, 1/5000) overnight at 4°C. Later, The membranes were washed three times with TBST for 10 min each, followed by incubation with horseradish peroxidase‐conjugated secondary antibodies (Boster, Wuhan, China) at a dilution of 1:5000 for 2 h at room temperature. After washing the membranes three more times with TBST for 10 min each, the protein bands were detected using an Enhanced Chemiluminescence Western blotting Substrate (Solarbio, Beijing, China) and visualized with a chemiluminescence imaging system (Bio‐Rad, California, USA).

### 4.5 Cell uptake analysis

In vitro cellular uptake of LXY30‐Exos was investigated in A549 cells following the previously reported methods with minor changes.^46^ A549 cells (3 × 10^4^ cells) were seeded into 12‐well plates and were co‐cultured with 50 μg LXY30‐Exos labelled with Dil (Ruitaibio, Beijing, China) for 24 h. Followed by the co‐culture, the cells were rinsed, fixed and DAPI (Sigma‐Aldrich) stained for 5 min. Photomicrographs were captured by a fluorescence microscope (Olympus, Tokyo, Japan).

### 4.6 Flow cytometry

A549 cells were cultured in six‐well plates at 60% fusion for 24 h. Before the cells were co‐cultured with LXY30‐Exos, we replaced the medium with an FBS‐free medium (Thermo Fisher Scientific). Then, green fluorescent protein (GFP)‐labelled 50 μg LXY30‐Exos was co‐cultured with A549 cells. After co‐culture, the cells were suspended with 5% trypsin (Sigma‐Aldrich) and concentrated to about 1 million cells per 50 μL. The GFP‐positive cell rate was evaluated using flow cytometry.

### 4.7 Tumour xenografts

Female athymic nude mice (Six‐week‐old) were acquired from Cyagen (Suzhou, China). A549 cells (5 × 10^6^ cells) were subcutaneously implanted into the right abdomen of animals.^21^ When the tumour volume reached 100 mm^3^, PKH67‐labelled Exos (250 μL, 1 mg/kg) and PKH67‐labelled LXY30‐Exos (250 μL, 1 mg/kg) were intraperitoneally injected into mice.^37^ In vivo, imaging analysis was performed 21 days after injection. All the mice were euthanized, and tissues including tumours, spleen, liver, kidneys and lungs were harvested for imaging.^20^

### 4.8 Immunofluorescence staining

To investigate the distribution of PKH67‐labelled LXY30‐Exos in mice, the different tissues including the tumours, spleen, liver, kidneys and lungs were isolated. Images were captured and preserved under an immunofluorescence microscope (Olympus). The fluorescence intensities in photomicrographs were measured by Image‐Pro Plus.^47^

### 4.9 Clinical samples

A total of 30 NSCLC patients were enrolled. Patients with NSCLC have not received any prior treatment, including chemotherapy, radiation, targeted therapy or surgical resection. The study was approved by the ethics committee of the affiliated hospital of Nantong University. Patients were informed of the necessary information about the study and signed written informed consent.

### 4.10 Quantitative real‐time PCR

Total RNA was extracted using the Trizol method (Beyotime Biotechnology). Then the quality of total RNAs was evaluated by a NanoDrop1000 spectrophotometer (NanoDrop Technologies, Wilmington, USA). MiR‐30c, miR‐181b and miR‐613 expressions in tissues, cells and Exos were assessed using a TaqMan microRNA reverse transcription and microRNA assay kit (Applied Biosystems, California, USA). We use 7500 Real‐Time PCR Systems (Applied Biosystems). The relative levels of miR‐30c, miR‐181b and miR‐613 were evaluated using the 2^−ΔΔCt^ method implementing U6 as internal control. A list of all used primers is given in Table 1.

**TABLE 1:** The sequences of all primers used in qRT‐PCR.

Gene name	Primer sequence (5′‐3′)
miR‐30c	Forward: ACACTCCAGCTGGGTGTAAACATCCTACACTCT
	Reverse: CTCAACTGGTGTCGTGGA
miR‐181b	Forward: GCGGATCATTCATTGCTGTCG
	Reverse: GTGCAGGGTCCGAGGT
miR‐613	Forward: GTGAGTGCGTTTCCAAGTGT
	Reverse: TGAGTGGCAAAGAAGGAACAT
U6	Forward: CTCGCTTCGGCAGCACATA
	Reverse: GTGCAGGGTCCGAGGT

### 4.11 Preparation of LXY30‐Exos‐encapsulated miRNAs

The encapsulation of miR‐30c, miR‐181b and miR‐613 was conducted using a commercial kit (ExoFectin® sRNA‐into‐Exosome Kit, Shanghai, China) following the previously reported methods.^48^ MiR‐30c, miR‐181b and miR‐613 were procured from Sangon Biotech (Shanghai, China). The encapsulation of miR‐30c, miR‐181b and miR‐613 were prepared with an ExoFectin® facilitated methodology. The mixture of 5 μL TransExo reagent and 25 μL TransExo buffer was supplemented with 5 μM miR‐30c, miR‐181b or miR‐613 and incubated at room temperature for 5 min. Then, 50 μg LXY30‐Exos were added to the solution and incubated overnight at 37°C. Later, 10 μL RNase (1 mg/mL) was added to remove un‐transfected miR‐30c, miR‐181b or miR‐613 for 10 min. LXY30‐Exos‐encapsulated miR‐30c (Exo‐miR‐30c), LXY30‐Exos‐encapsulated miR‐181b (Exo‐miR‐181b) or LXY30‐Exos‐encapsulated miR‐613 (Exo‐miR‐613) were sufficiently characterized before the subsequent assays.

### 4.12 Cell viability assay

The proliferation of NSCLC cells was assessed using a cell counting kit‐8 assay. First, A549 cells were co‐cultured with Exo‐miR‐30c (50 μg), Exo‐miR‐181b (50 μg) or Exo‐miR‐613 (50 μg) for 24 h. Then, the cells (3 × 10^3^) were seeded into 96‐well plates and added CCK‐8 reagent (Sigma‐Aldrich), incubated at 37°C for 1 h. Lastly, the absorbance of each well was recorded at 450 nm using a Synergy H4 Hybrid Reader (Thermo Fisher Scientific) at different time intervals 0, 24, 48 and 72 h to determine cell proliferation.

### 4.13 Colony formation assay

A549 cells (1 × 10^3^) were co‐cultured with Exo‐miR‐30c (50 μg), Exo‐miR‐181b (50 μg) or Exo‐miR‐613 (50 μg). Twenty‐four hours later, A549 cells were grown in six‐well plates. Ten days later, we fixed the colonies by 4% PFA (Sigma‐Aldrich) and stained with crystal violet dye (Sigma‐Aldrich). The colonies were counted under a microscope (Olympus).

### 4.14 Cell migration analysis

For wound healing, A549 cells were co‐cultured with Exo‐miR‐30c (50 μg), Exo‐miR‐181b (50 μg) or Exo‐miR‐613 (50 μg) for 24 h. The cells (2 × 10^5^) were put in six‐well plates. The scratch wound was generated with a 200 μL sterile straw tip, and the floating cells were removed by washing with PBS. At 0 h and 24 h after the scratches, the scratches were photographed by an inverted microscope (Olympus).

Transwell assays were used to assess A549 cell migration. A549 cells were co‐cultured with Exo‐miR‐30c (50 μg), Exo‐miR‐181b (50 μg) or Exo‐miR‐613 (50 μg) for 24 h. A549 cells (2 × 10^4^) were put in the upper chamber with an insert (Millipore). The lower chamber was filled with complete media. After 24 h, cells were fixed, stained, counted and photographed for preservation.

### 4.15 Cell cycle and apoptosis assays

A549 cells (3 × 10^4^ cells) were co‐cultured with Exo‐miR‐30c (50 μg), Exo‐miR‐181b (50 μg) or Exo‐miR‐613 (50 μg) for 24 h. After that, the cells were analysed for cell cycle progression using a Cell Cycle Assay Kit (Abcam, Cambridge, UK) following the manufacturers' standard procedure. Following harvesting, the cells were fixed and stained in darkness with propidium iodide (PI) at a concentration of 50 μg/mL for 25 min. The percentage of cells in different phases was determined by flow cytometry.

To ascertain apoptosis induction, we digested the cells and stained them using an Annexin V/PI Apoptosis Detection Kit (Solarbio, Beijing, China), and A549 cell apoptosis was assessed using flow cytometry.

### 4.16 In vivo assay

The effects of Exo‐miR‐30c, Exo‐miR‐181b or Exo‐miR‐613 on the growth of NSCLC tumours were further assessed. Mice (*n* = 12, 3/group) were grouped: Exo‐NC, Exo‐miR‐30c, Exo‐miR‐181b and Exo‐miR‐613. The tumour xenograft mouse model was constructed following the above‐described methods. When the tumour volume reached 100 mm^3^, Exo‐NC, Exo‐miR‐30c, Exo‐miR‐181b or Exo‐miR‐613 (200 μg/mouse for 10 times in total) was intratumorally injected into the mice every 2 days.^9^ After 2 weeks, the tumour weight was weighed, and the tumour growth curve was plotted. Tumour volume = (length× width^2^)/2.

### 4.17 Haematoxylin–eosin staining

In order to evaluate the safety of Exo‐miR‐30c, Exo‐miR‐181b or Exo‐miR‐613 in vivo, pathological changes in spleen, liver, lung, heart and kidney tissues were evaluated using haematoxylin–eosin staining. The isolated mouse liver, spleen, lung, kidney and heart tissues were fixed with 4% PFA (Sigma‐Aldrich) and embedded in paraffin. After making 5 μm thick continuous sections, the pathological analysis of the above tissues was conducted using the haematoxylin–eosin Staining Kit (Solarbio) according to the manufacturer's standard protocol.

### 4.18 Immunohistochemical assay

The expression of Ki‐67, E‐cadherin and N‐cadherin in NSCLC tissue was tested using immunohistochemical assays. NSCLC tissues were fixed with 4% PFA (Sigma‐Aldrich), embedded in paraffin, and carved into sections with 5 μm thickness. All tissue sections were incubated with primary antibodies containing anti‐Ki‐67 (ab15580, 3 μg/mL), anti‐E‐cadherin (ab231303, 1 μg/mL) and anti‐N‐cadherin (ab207608, 1/500) at 4°C overnight. All the sections were coloured with DAB (Sigma‐Aldrich) and redyed with haematoxylin (Solarbio). All images are captured and saved using a light microscope (Olympus).

### 4.19 Serum biochemistry and routine blood tests

Serum samples from mice were harvested. The AST, ALT, BUN, CRE and T‐Bil contents in mouse serum were respectively determined using AST Enzyme‐linked Immunosorbent Assay (ELISA) Kit (Abcam), ALT ELISA Kit (Abcam), CRE ELISA Kit (Jianglaibio), T‐Bil ELISA Kit (Institute, Shanghai, China) following the manufacturer's standard protocol. For the WBC analysis, the blood samples were collected and aliquoted into EDTA‐2Na containing blood collection tubes. Total WBC was analysed through a veterinary multi‐parameter haematology analyser (Sysmex, Japan).

### 4.20 Statistical analyses

All the data shown are mean ± SD of independent assays performed in triplicate. The statistical evaluations were performed using SPSS 19.0 software (Chicago, USA). The Student's *t*‐test was performed for comparisons of two separate groups. One‐way ANOVA was conducted for comparisons of multiple independent groups. *p*‐values <0.05 were considered statistically significant.

## 5 CONCLUSIONS

Our study confirms that BMSC‐Exos with LXY30 peptide modification specifically targets NSCLC tumours. Furthermore, LXY30‐Exos‐encapsulated miR‐30c, LXY30‐Exos‐encapsulated miR‐181b or LXY30‐Exos‐encapsulated miR‐613 repressed NSCLC growth in both in vitro and in vivo models. Meanwhile, the safety of engineered Exos was tentatively confirmed. The completion of this study might provide a novel approach for NSCLC therapy.

## AUTHOR CONTRIBUTIONS

**Mingjun Yang:** Conceptualization (equal); formal analysis (equal); writing – review and editing (equal). **Wen Zhou:** Data curation (equal); formal analysis (equal); investigation (equal); methodology (equal). **Xiao Han:** Data curation (equal); formal analysis (equal); investigation (equal); methodology (equal). **Mingming Xu:** Data curation (equal); formal analysis (equal); investigation (equal); methodology (equal). **Zhipeng Wang:** Software (equal); validation (equal); visualization (equal). **Min Shi:** Visualization (equal); writing – original draft (equal). **Yanyan Shi:** Writing – original draft (equal). **Yunchi Yu:** Software (equal); validation (equal); visualization (equal).

## FUNDING INFORMATION

This work was supported by the Nantong Science and Technology Planning Project (No. MS12021095).

## CONFLICT OF INTEREST STATEMENT

The authors declare that they have no conflicts of interest.

## Supporting information


Figure S1.

## Data Availability

The raw data supporting the conclusions of this manuscript will be made available by the authors, without undue reservation, to any qualified researcher.
