# Supplementary figures and images for: Modified bone marrow mesenchymal stem cells derived exosomes loaded with MiRNA ameliorates non‐small cell lung cancer
Source: J Cell Mol Med. 2024 Sep 25;28(18):e70115. doi: 10.1111/jcmm.70115 (PMC11423648; doi:10.1111/jcmm.70115)

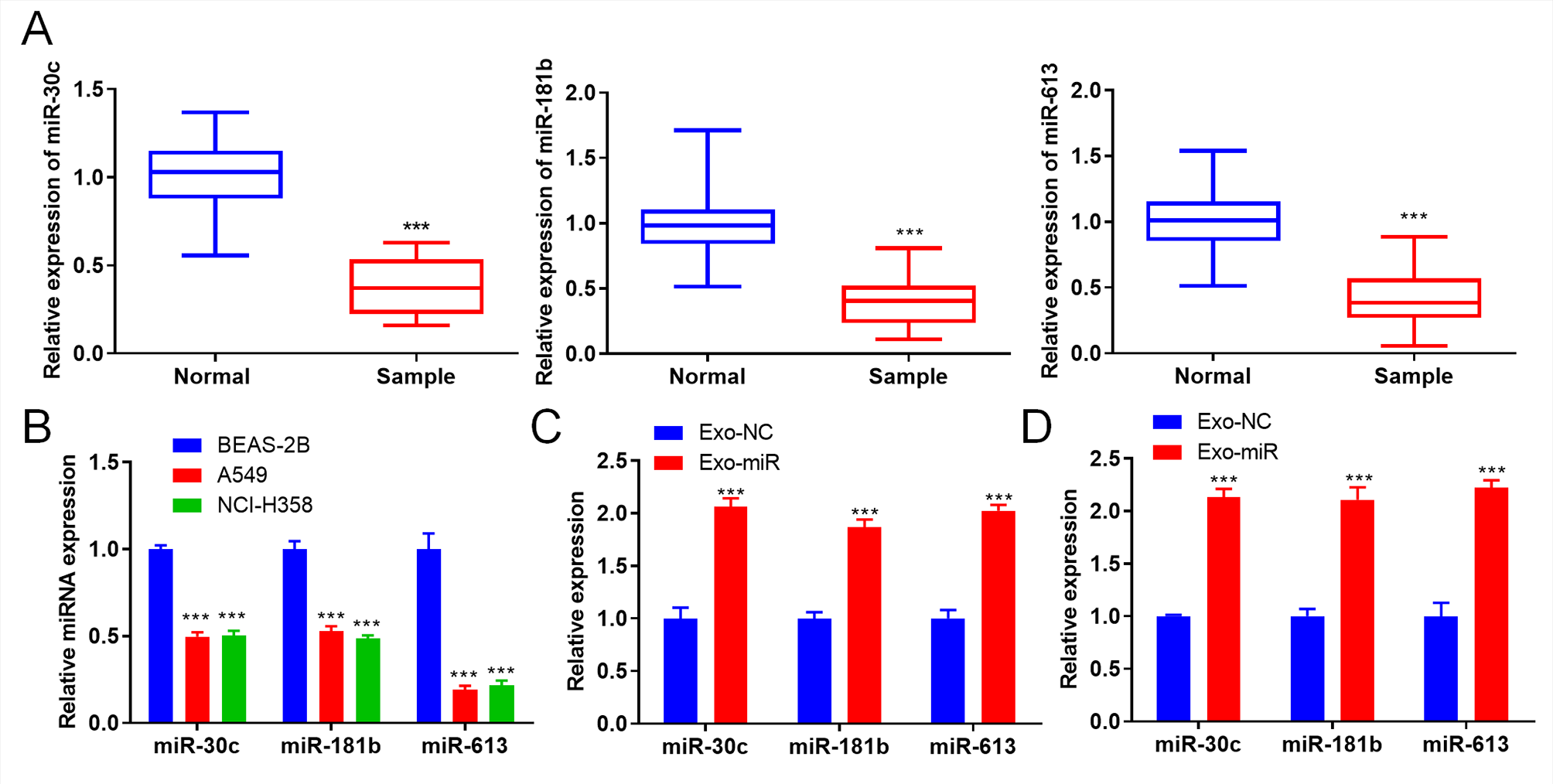

Supplement: Supplementary file 1 — Figure S1. [file JCMM-28-e70115-s001.tif]
